# Changes in Population-Level Consumption of Taxed and Non-Taxed Sugar-Sweetened Beverages (SSB) after Implementation of SSB Excise Tax in Thailand: A Prospective Cohort Study

**DOI:** 10.3390/nu12113294

**Published:** 2020-10-27

**Authors:** Sirinya Phulkerd, Natjera Thongcharoenchupong, Aphichat Chamratrithirong, Rossarin Soottipong Gray, Piyada Prasertsom

**Affiliations:** 1 Institute for Population and Social Research, Mahidol University, Nakhon Pathom 73170, Thailand; birt_redmachine@hotmail.com (N.T.); aphichat.cha@mahidol.ac.th (A.C.); rossarin.gra@mahidol.ac.th (R.S.G.); 2 Bureau of Dental Health, Department of Health, Ministry of Public Health, Nonthaburi 11000, Thailand; pprasertsom@gmail.com

**Keywords:** sugar-sweetened beverage, excise tax, fiscal policy, Thailand, obesity, non-communicable diseases

## Abstract

Background: Thailand has implemented a sugar-sweetened beverage (SSB) excise tax since September 2017, but questions remain about changes in individual-level SSB consumption after tax implementation. This study aimed to analyze the change in taxed and non-taxed SSB consumption in the Thai population. Method: This cohort study was carried out in 2018 and 2019. A total of 5594 persons who participated in both years were included in the analysis. Descriptive and *t*-test analyses were carried out in order to identify the significance of the difference between taxed and non-taxed SSB consumption in 2018 and 2019. Results: A lower mean ratio of average taxed SSBs consumed to average non-taxed SSBs consumed was found to be statistically significant (*p* = 0.001). That is, the decline in taxed SSB consumption is significantly greater than that of the non-taxed consumption. The greater reduction in taxed SSB consumption than the non-taxed SSB consumption was found to be significant among males, older persons, the lower-income population, and the unemployed. Conclusions: Overall, the current SSB tax has the capacity to successfully discourage consumption of SSBs with high sugar level. The decline in SSB consumption is, however, not found across all socioeconomic groups. Thresholds for SSB-tiered tax structure may have to be reviewed.

## 1. Introduction

Unhealthy diet is a significant public-health concern worldwide, given its strong linkage with overweight and obesity and non-communicable diseases (NCDs) and is associated with higher health costs and reduced productivity [1]. Thailand is like many developing and developed countries that experience high prevalence of overweight and obesity across population groups. National evidence shows that the prevalence of overweight and obesity reached by 37.5% among adults in 2014 [2] and 9.7% among children in 2008–2009 [3], and these rates have gradually increased over the past decade.

Increasing sugar intake contributes to unhealthy diet and is associated with weight gain [4]. In addition, people with high intake of sugar-sweetened beverages (SSBs) are more likely to have an increased risk of NCDs than those with a low SSB intake [5,6,7]. In Thailand, average daily sugar intake is 25 teaspoons or 100 grams [8], which is four times higher than the recommended level (below 25 grams or 6 teaspoons per day), according to World Health Organization’s recommendation [9]. Frequency of SSB intake is high among children, as about 31% consumed a SSB at least four days a week [10]. There are also rural/urban differences among children. Children in urban areas had more frequent SSB consumption (36.5%) than those in rural areas (28.4%) [10]. Similar to the adult population, urban adults consumed SSBs more frequently than rural adults [10].

Using pricing policies, such as excise tax increases on SSBs, is one of the recommended actions to promote healthy diet for preventing obesity and NCDs [1]. The introduction of a SSB tax in Thailand was largely influenced by the nongovernmental sector, such as the Sweet Enough Network, Thai Health Promotion Foundation, and International Health Policy Program, in collaboration with government agencies, especially the Department of Health and Excise Department [11]. At that time, the excise tax rate structure of SSBs under the Excise Tax Act B.E. 2527 (1984) mainly focused on raising government revenue and increasing income of local food agriculturists. Sugar-added beverages were taxed at a lower rate than non-sugar added beverages. Criteria were set for fruit and vegetable juices, based on the ratio of juice-to-total-volume, and tea and coffee were tax-exempt. Accordingly, several types of SSBs were sold at a lower price than non-SSBs. Therefore, since 2008, the aforementioned organizations had advocated for excise tax reform to contribute to healthier consumer behavior, while generating increased revenue.

On 16 September 2017, taxation of SSBs by sugar content came into force in Thailand. The SSB taxation was adopted by the Excise Department, Ministry of Finance, through amendments to laws relating to excise tax collection by merging the initial seven of such acts, which include the Excise Tax Act B.E. 2527 (1984), the Excise Tax Code Act B.E. 2527 (1984), the Excise Tax Allocation Act B.E. 2527 (1984), the Liquor Act B.E. 2493 (1950), the Liquor Tax Allocation Act B.E. 2527 (1984), the Tobacco Act B.E. 2509 (1966), and the Playing Cards Act B.E. 2486 (1943) into the Excise Tax Act B.E. 2560 (2017) [11].

The Excise Department implemented a tiered tax approach, taxing beverages at different amounts, depending on their sugar content (grams (g) of sugar per 100 milliliters (mL)) [11]. The tax table categorizes sugar content in SSBs, based on six levels: less than 6 g, 6–8 g, > 8–10 g, > 10–14 g, > 14–18 g, and >18 g. The SSB products that contain less than 6 g sugar per 100 mL are exempt from the tax, while those products containing 6 g or higher sugar per 100 mL are taxed at a higher rate. In addition, the tax rate increases every two years. However, the Excise Department offered a grace period to help beverage manufacturers gradually lower sugar content of their products to meet the tax-tier thresholds if they chose to do so [11]. The grace period is divided into three phases of two years each [11], and was granted to the manufacturers before the new rates were applied. Thailand has already completed the first phase of the SSB tax and is in the second phase, which came into effect on 1 October 2019.

SSB taxation has drawn growing interest globally, and some countries have already implemented the SSB tax, such as Mexico, Colombia, Hungary, Brunei, Malaysia, France, Finland, South Africa, and some states in the United States [12]. Some countries have shown a decline in SSB consumption and purchase after implementation of the SSB tax. A recent cohort study in Mexico found an association between implementation of the Mexican SSB tax and a reduction in the probability of consuming SSBs among employees from a healthcare provider [13]. Another study in the same country showed a decrease in purchases of taxed SSBs by 8.2% over the two years after tax implementation (in 2014 and in 2015) [14].

Thailand has implemented the SSB tax since September 2017, but questions remain about the change in post-tax individual-level consumption. The implicit assumption behind the tax policy is that reducing consumption of sugars [15] has positive health effects. Evidence about the change in SSB consumption after the tax implementation among the Thai population will allow policymakers and other stakeholders to understand the utility of this tax policy, and help them make timely decisions in improving population diet and health. Such evidence is not available to date.

Accordingly, this study aimed to analyze the change in SSB consumption in the Thai population by sociodemographic groups and by taxed and non-taxed SSB products, using data from a nationally representative longitudinal cohort survey of the Thai population, in 2018 and 2019.

## 2. Materials and Methods

### 2.1. Study Design and Population

This study used data from a monitoring survey of SSB consumption that was embedded in a longitudinal, population-based household survey of fruit and vegetable consumption in the Thai population [16]. The survey provides the most recent available SSB consumption dataset, and aimed to monitor consumption of the most commonly consumed SSB products after SSB taxation was put into effect. The data collection occurred at baseline-Wave 1 (in 2018) and Wave 2 (in 2019).

A multi-stage sampling design was used to obtain a nationally representative sample of persons age six years or older, in a survey of households in the four geographic regions of Thailand (North, Central, Northeast and South) and Bangkok. The sampling design was done by the National Statistical Office (NSO) of the Ministry of Information and Communication Technology (which is also responsible for the national census). The NSO conducts periodic household surveys to obtain national data on socioeconomic characteristics, usage of information technology, and other issues of interest.

The geography of the sampling frame is hierarchically nested: geographic region, province, district, enumeration area (EA), and household. Within each region, two provinces were selected, and then districts within each province were sampled. From these districts, EA and households were selected as sampling units (20 households in each EA), designated by the NSO. All eligible households within sampled EAs were approached for participation in the study.

Mapping and household listing in each EA were prepared by the NSO. The target households were contacted through local sub-district office and/or village leaders. The data were collected from all the household members who were present. If the data collector failed to reach a household member after three visits, that member was excluded; a replacement was not taken.

This study was conducted according to the guidelines laid down in the Declaration of Helsinki, and all procedures involving research study participants were approved by the Institutional Review Board (IRB) of the Institute for Population and Social Research of Mahidol University (COA. No. 2018/05-166 and COA. No. 2019/06-200). Written informed consent was obtained from all subjects.

### 2.2. Data Collection

Data were collected in two rounds (2018 and 2019), from the same households. All household members age six years or older were eligible to participate in the survey. Household members who did not participate in Wave 1 and Wave 2 were either sick or did not stay in their house during the survey. The first and second waves of the survey were respectively conducted during May to December 2018, and June 2019 to January 2020. The one-month late start of the second wave was due to a delay in proposal approval process.

In the first survey round, a total of 3720 households were sampled, of which 3670 successfully completed the survey (response rate of 98.7%) (Table 1). A total of 7957 participants age six years or older completed the survey. In the second round, a total of 3333 households participated in the survey (response rate of 90.8%), with a total of 7847 participants who completed the survey. All of the interviewees who participated in both waves of the survey were included in analysis. Of those, 5594 successfully completed the survey.

Data collection was done, face-to-face, by trained interviewers, using a structured questionnaire. Due to the complex questionnaire used in this study, interviewers were selected on the basis of their level of education and previous experience in conducting relatively complex interviews. Written informed consent form was obtained from each participant before the interview.

### 2.3. Study Variables

#### 2.3.1. Outcome Variables

To assess current SSB intake, participants were asked how often (number of days), if ever, they had consumed each of specific taxed and non-taxed SSB type in a typical week, and how much (number of servings) the participant consumed on each day. Servings were determined based on the response after showing pictures of each of SSB items for one serving size. Responses to the frequency and quantity were multiplied, to calculate the average amount consumed in the past week.

The study considered those products sold in a can, bottle, box, or glass containing up to 650 mL as one serving, regardless of what is stated on the product as a serving size. Products with a 1 liter size were excluded from the study because it was difficult to quantify individual intake, as these products are often shared or consumed gradually over time. The study focused on the most commonly consumed SSB products among Thai people and recorded its sugar content per serving (Table 2), according to data from the 2017 Food Labeling Monitoring Survey [17], conducted by the Sweet Enough Network—a non-profit and non-governmental organization in collaboration with Dental Health Bureau of the Department of Health, Ministry of Public Health (MOPH), and the Thai Health Promotion Foundation (ThaiHealth).

#### 2.3.2. Independent Variables

The following independent variables that were considered potentially related to SSB consumption were included in the analysis: sex, age, marital status, place of residence, occupation, personal income, and educational attainment. Sex was composed of two categories: male and female. Age was categorized into three age groups, namely 6–14, 15–59, and 60 years or over. Marital status was divided into three groups: married, single, and widowed/divorced/separated. Place of residence was dichotomized into rural or urban area. Educational attainment was categorized as follows: no formal education, primary school, secondary school, and Bachelor’s or higher degree. Personal income (baht per month) was divided into four groups: no income, lower than 10,000, 10,000–29,999, and 30,000 or above. Current occupation comprised six groups: unemployed (including being not in labor force), government employee, company-hired worker, own business, wage labor, and farmer.

#### 2.3.3. Data Analysis

Descriptive statistics were used in describing changes in SSB consumption of the sample in 2018 and 2019. The distribution of sociodemographic characteristics of people who consumed taxed SSB products and of people who consumed non-taxed SSB products was compared. A *t*-test was used to determine if there was a significant difference between the means of the ratio of average taxed SSBs consumed to average non-taxed SSBs consumed in Thai population. The threshold for statistical significance for all analyses was set at *p* = 0.05.

## 3. Results

Overall, a reduction in consumption of taxed SSBs and non-taxed SSBs was observed in this national sample of the Thai population, between 2018 and 2019 (Table 3). Average daily SSB consumption declined from 474.0 mL in 2018 to 453.8 mL in 2019, or a 2.5% decrease. All age groups reported reduced SSB consumption. Thais age 6–14 years had the highest reduction (−4.9%) compared to younger (−3.4%) and older (−0.5%) adults.

When comparing the two groups, we noted that there was a reduction in both taxed and non-taxed SSBs consumed between 2018 and 2019, with −2.8% and −2.0%, respectively. The highest reduction in consumption of taxed SSBs was found in persons age 60 years or older (−7.2%), while the highest reduction in consumption of non-taxed SSBs was found in those age 15–59 years (−3.2%).

Among the taxed products, carbonated drinks (325 mL can) experienced the largest reduction in consumption (−17.7%). This is followed by herbal drinks (400 mL bottle) and non-100% fruit juice drinks (200 mL box). Conversely, an increase was observed in consumption of other SSB products, especially non-0% sugar green tea (500 mL bottle), which posted the largest increase (+13.8). By age group, consumption of carbonated drinks (325 mL can) declined most among children (−26.0%) and younger adults (−9.5%). The highest reduction in consumption of herbal drinks (400 mL bottle) was observed among older persons (−29.3%). The highest increase in consumption of non-0% sugar green tea (500 mL bottle) was found among younger adults.

Among the non-taxed products, the largest reduction in consumption was for flavored milk (200 mL bottle) (−9.6%), followed by flavored milk (200 mL box) and sour milk / yogurt (180 mL box). Conversely, sour milk/yogurt (100 mL bottle), fresh-made herbal drinks (500 mL glass), and fresh-made iced tea/coffee (650 mL glass) showed an increasing trend in consumption. By age group, the decrease was most pronounced among children and older persons, with −30.3% and −7.2%, respectively. Younger adults reported the largest decrease in consumption of flavored milk (200 mL box) (−24.2%). Sour milk/yogurt (100 mL bottle) was increasingly prevalent among older persons. The *t*-test analysis was applied to assess the difference in the mean ratio of average taxed SSBs consumed to average non-taxed SSBs consumed in 2018 and 2019 (Table 4). A decreased ratio from 2018 to 2019 was observed. The difference between the mean ratio in 2018 and 2019 was found to be statistically significant (*p* = 0.001). That is to say, the decline in taxed SSBs consumption was significantly greater than that of non-taxed SSBs consumption.

Table 4 also shows a significant difference in the mean ratio of average taxed SSBs consumed to average non-taxed SSBs consumed, disaggregated by sociodemographic characteristics in 2018 and 2019. The results indicate that there was a statistically significant difference in the mean ratio between 2018 and 2019 in males, older persons, people who were single or married, people living in rural and urban areas, people living in Bangkok, people who completed primary and secondary school, people with salary lower than 10,000 baht per month, and the unemployed. The most significant difference between the mean ratios in 2018 and 2019 was found in older persons (*p* < 0.001), followed by people living in Bangkok (*p* = 0.004), and males (*p* = 0.005). Among income groups, significant mean ratio differences were found only in people with a monthly income lower than 10,000 baht (*p* = 0.01). Significant mean ratio differences were also only found among the unemployed (*p* = 0.03) while this study found no significant mean ratio difference for other employment categories.

## 4. Discussion

This study provides the first population-based evidence in Thailand and Southeast Asia to assess the changes in population-level SSB consumption after the excise tax implementation (which increased with sugar-by-volume). This national study observed changes in consumption of taxed and non-taxed SSBs in the population age six years or older after two years of implementation of the SSB tax. The comparison between the ratio of the taxed to the non-taxed SSBs consumed over time shows significant reductions in the consumption of the taxed group of SSB. These reductions are likely to have been caused by the SSB excise tax. The impact of SSB tax on SSB consumption was also observed in other countries. In Spain, following the tax’s introduction, the prevalence of regular consumers of taxed beverages decreased by 35.2% in Barcelona and increased by 6.2% in Madrid [18]. In Mexico, the SSB tax was found to be associated with a reduction in the probability of consuming soft drinks in the cohort of employees, from a healthcare provider, after three years of implementation [13].

For taxed beverages, the study found an average decline of −2.8% in consumption over the two, post-tax years. The reduction was largest for carbonated and herbal drinks. This is not surprising, as these two products are among those with the highest sugar content and, thus, would have experienced the largest increase in retail price. Results of a monitoring survey on price and sugar content of the SSBs before and after tax implementation showed that the tax was reflected in increased price for SSBs (except for energy drinks), and was highest for carbonated beverages (60% increase in price) [19]. It is reasonable to assume that beverage distributors passed the tax on to retailers, who, in turn, passed the increased cost on to consumers, thus depressing consumer purchases and, hence, consumption. Public announcements by some carbonated beverage companies confirmed that sales and profits underperformed after the tax was introduced [20,21]. After the announcement of the tax, some companies announced their intention to gradually reformulate their products, to reduce the sugar content to avoid the tax [22,23].

Consumption of some taxed SSBs had an average increase over the two post-tax years. In particular, consumption change of non-0% sugar green tea (500 mL bottle) increased the most (13.8%). Compared to other drinks, the green tea drinks are lowest in sugar content and, thus, are the least affected by the tax. Accordingly, the tea drinks companies were more likely to bear the burden of the tax in order to keep their product price stable. It is possible that the green tea companies saw the tax as an opportunity to gain market share among middle- and low-income consumers. Currently, their marketing campaigns mainly focus on communicating the superior health benefits of green tea over other common drinks, like sweetened sodas and coffee [24]. Some companies also ran “lucky draw” campaigns, with prizes ranging from free drinks to cars or one million baht [24], and these campaigns were particularly effective among lower-income consumers. Although the tax may offer added incentives for product reformulation and greater incentives for behavior change for most of the SSBs, the tax may not work with some particular beverages. Non-taxed policy (e.g., food marketing) should be designed, along with the consumption tax policy, to enhance the overall effect of the tax on healthier diets among the Thai population.

This study observed an increase in consumption of energy drinks (4.6%), which are high in sugar and have the second highest market share of the soft drinks market in Thailand, after carbonated drinks [25]. The introduction of the added tax increased the cost by of 0.32–0.90 baht for each bottle of energy drink sold. Thus, the increase in consumption over the study period is surprising, since the main consumers of energy drinks are laborers and agricultural workers, who are sensitive to changes in economic circumstances. However, it is possible that the sales were boosted by the producers of energy drinks, which maintained sales volume by holding the retail price of their goods stable, according to marketing analysis [26]. In addition, consumption may have been stimulated by some government policies, such as increases to the national minimum wage [27], and welfare card scheme with extra financial support to lower-income earners and wage laborers [28], which can have a positive impact on the spending power of lower-income groups. This growth in consumption is consistent with the trends in sales of energy drinks between 2018 and 2019. The sales of these SSBs in 2019 continued to grow, though not at a high rate, e.g., by 0.7% from 2018 (from 415.7 to 418.7 million liters) [25]. Although there is inconsistency between tax policy (for health promotion) and social welfare policy (for poverty eradication), the announcement of the second-round tax increase by the Excise Department (effective in October 2019) has speeded up the decision of many producers of SSBs (including energy drinks) to reformulate their products (i.e., to reduce sugar content)[29]. Moreover, since the tax is progressive, there should be greater sugar-by-volume reductions in the sweetest SSBs.

For non-taxed SSBs, the study found a drop in consumption of −2.0% over the two post-tax years. The highest reduction was observed for milk products. This effect is likely beyond the legislative intent. One possible reason is that there was an indirect (spillover) effect of the tax policy on consumer behavior. The tax may create a positive spillover effect that occurs when changes in one behavior influence changes in linked behaviors [30]. The SSB tax could influence consumers to think that tax enforcement applies to other sugary products, as well. The incentive for a consumer’s first-time purchase of the taxed product with a higher price could unintentionally create a negative experience for second-time purchase of other sugary products in order to avoid spending more money. This spillover effect can accelerate non-taxed producer’s efforts to reduce sugar content in their products, and that will give the producers a competitive advantage in the SSB market without paying any extra taxes. Likewise, spillover effects of eco-labeled shopping combined with positive verbal feedback or economic incentives spilled over to other pro-environmental actions, such as turning off the lights when leaving a room [31]. However, spillover effects can also affect behaviors in a negative direction, such as drinking more local or homemade drinks, which can also be high in sugar, such as tea, coffee, and herbal drinks. Thus, positive and negative spillover effects can impact on the net effect of the policy. This study suggests that a better understanding of spillover effects in consumer behavior and taxation is needed.

The findings from this study suggest that SSB tax policy was effective in deterring SSB consumption among older consumers, the lower-income population, and the unemployed. This is not surprising, as it could be the result of the direct income effect. This supports the tax intent that is financially regressive by imposing a larger burden on lower-income people [32]. That result is consistent with a study by Lope and Fantuzzi, suggesting that SSB tax—on carbonated drinks in particular—has a more significant impact on low-income consumers [33]. However, the study indicated that younger and male groups are among the most affected. That finding contradicts the present study which found that older persons were the most affected group. This might be due to characteristics of Thai persons age 60 years or older, of whom about one-third were financially dependent on their child [34], and one-fourth lived below the poverty line in 2018 (2710 baht per month).

By contrast, the tax policy seems not to work among the higher-educated and those with more stable income. These groups can probably (still) afford the higher price of these SSBs (i.e., inelastic demand). It could also partly be explained by size of the tax. Previous research demonstrates that the largest tax that would cause higher-income households to pay the largest share of the tax (30%), compared to the lowest- and middle-income quartiles, was a 40% price increase on SSBs [35]. Thus, further analyses are needed to examine tax thresholds and benefit levels on individual consumption and purchases by beverage category and sociodemographic characteristics. That information would help inform policymakers about potential thresholds for a SSB-tiered tax structure in different population groups in Thailand.

This study found a significant reduction of SSB consumption among people living in Bangkok, as compared to other geographic regions of Thailand. Due to modern daily living and a busy hectic lifestyle, people living in a large city like Bangkok likely have high exposure to ready-to-eat food and beverage that are sold for instant consumption, such as SSBs, and that is associated with NCDs, such as type 2 diabetes [36]. The big-city dwellers are also more likely to rely on a larger number of platforms for news and information via a range of digital channels. It is possible that these people were more aware of the SSB excise tax during the pre- and post-tax years through various media. When people become more aware about a progressive tax and its effect on SSB price change, that could influence their purchase decisions and, ultimately, consumption. Accordingly, there is a need for public communication on SSB tax with greater dissemination in other provinces and rural settings.

The novelty and strength of this study deserve attention. First, this was a population-based prospective study, with high participation rates, and virtually complete cohort follow-up, minimizing many sources of bias inherent to case-control studies. This study went further than examining only the amount of taxed SSB intake; it also considered non-taxed SSB consumption. The study also analyzed SSB intake by beverage category and by sociodemographic characteristics.

That said, several limitations are also acknowledged. As with any observational study, the results are based on self-reported data in which the accuracy of SSB intake from questionnaires is variable and prone to participant recall bias. The study was further subject to a limitation from lack of information on changes in store-bought purchases and consumption habits, especially substitution behaviors. Combining these data with the data collected would help monitor change overtime and the effect on the intake of sugar in the population. Regression analysis of factors associated with taxed SSB consumption is also needed. Future analysis of the manufacturers’ responses to the tax for both taxed and non-taxed products, such as reduction in total sugar or by replacing it with non-caloric sweeteners, and changes in price and portion size, is important to provide a better understanding of the longer-term implications of the SSB tax. Future analysis of health outcomes’ status, such as obesity and diet-related NCDs, is also important to see whether effects on health can be attributed to the tax.

## 5. Conclusions

At two years after its introduction, the Thai excise tax on SSBs has coincided with a significant decline in consumption of taxed SSBs. Future studies will have to assess whether this change is maintained over time, and measure not only the amounts consumed, but also the behavior of the population in store-bought purchases, product substitution, reformulation attempts of the SSB industry, and individual health and nutrition, in order to have a more accurate estimation of the tax’s impact, especially in different sociodemographic groups, such as those with higher-income and more education. This study suggests that the tax would be effective in decreasing SSB consumption, but could have only a hardly discernable effect on obesity and diet-related NCDs. Thus, a more comprehensive program, rather than a stand-alone tax policy on SSBs, is needed.

## Figures and Tables

**Table 1:** Number of sample household members and response rate of the survey in Waves 1 and 2.

Region	Sampled Households	Number of Households and Participants Interviewed				
		Wave 1		Wave 2		
		Households	Participants	Households	Participants	Participants (from Wave 1 Only)
Bangkok	620	619	1290	513	1146	854
Central	660	637	1377	581	1421	929
North	720	707	1417	707	1431	986
Northeast	920	908	1989	853	2059	1440
South	800	799	1884	734	1790	1385
**Total**	**3720**	**3670**	**7957**	**3333**	**7847**	**5594**

**Table 2:** Types of specific sugar-sweetened beverage (SSB) products in the study and sugar content of each product.

Product	Sugar Content (Grams) per Serving	Sugar Content (Grams) per 100 mL
**Taxed Group**		
1. Non-100% fruit juice (230 mL can)	24.33	10.58
2. Non-100% fruit juice (180 mL bottle)	19.04	10.58
3. Non-100% fruit juice (200 mL box)	21.16	10.58
4. Ready-to-drink coffee (180 mL can)	12.61	7.01
5. Non-0% sugar green tea (500 mL bottle)	36.95	7.39
6. Carbonated drinks (325 mL can)	40.88	12.58
7. Carbonated drinks (550 mL bottle)	69.19	12.58
8. Herbal drinks (400 mL bottle)	72.88	18.22
9. Energy drinks (100 mL bottle)	27.11	27.11
**Non-Taxed Group**		
10. Flavored milk (200 mL box)	39.18	19.59
11. Flavored milk (200 mL bottle)	39.18	19.59
12. Sour milk / yogurt (180 mL box)	18.23	10.12
13. Sour milk/yogurt (100 mL bottle)	10.13	10.13
14. Fresh-made iced tea/coffee (650 mL glass)	48.04	7.39
15. Fresh-made herbal drinks (500 mL glass)	91.10	18.22
16. Fresh-made smoothie juice (650 mL glass)	135.00	20.77

**Table 3:** Changes in consumption of sugar-sweetened beverages (mL/day) by taxed and non-taxed product and age group in 2018 and 2019.

SSB Product	Unit: mL per Day											
	Average Consumption in 2018	Average Consumption in 2019	% Change (+Increase –Decrease)	Age Group								
				6–14 Years			15–59 Years			60 Years or Over		
				2018	2019	% Change	2018	2019	% Change	2018	2019	% Change
**Taxed group**												
Non-100% fruit juice (230 mL can)	61.2	64.2	+(4.9)	66.5	60.3	−(9.3)	59.2	67.4	+(13.8)	55.2	58.9	+(6.7)
Non-100% fruit juice (180 mL bottle)	58.0	59.4	+(2.4)	63.1	57.4	−(9.0)	55.4	62.1	+(12.1)	53.9	56.4	+(4.6)
Non-100% fruit juice (200 mL box)	74.8	67.9	−(9.2)	77.9	62.4	−(19.9)	74.6	71.9	−(3.6)	70.7	62.0	−(12.3)
Ready-to-drink coffee (180 mL can)	112.3	121.2	+(7.9)	34.3	43.7	+(27.4)	112.3	121.7	+(8.4)	115.7	126.2	+(9.1)
Non-0% sugar green tea (500 mL bottle)	147.0	167.3	+(13.8)	154.5	175.9	+(13.8)	145.8	166.7	+(14.3)	112.3	99.9	−(11.0)
Carbonated drinks (325 mL can)	133.8	110.1	−(17.7)	135.1	100.0	−(26.0)	136.4	123.4	−(9.5)	77.0	82.3	+(6.9)
Carbonated drinks (550 mL bottle)	232.8	243.6	+(4.6)	217.5	237.5	+(9.2)	252.0	261.5	+(3.8)	178.4	173.0	−(3.0)
Herbal drinks (400 mL bottle)	125.4	112.8	−(10.0)	133.4	115.7	−(13.3)	116.2	112.5	−(3.2)	125.5	88.7	−(29.3)
Energy drinks (100 mL bottle)	54.3	56.8	+(4.6)	21.9	22.8	+(4.1)	55.6	53.6	−(3.6)	52.7	68.1	+(29.2)
**Total**	**283.6**	**275.8**	**−(2.8)**	**381.5**	**361.4**	**−(5.3)**	**282.2**	**280.8**	**−(0.5)**	**160.3**	**148.8**	**−(7.2)**
**Non-Taxed Group**												
Flavored milk (200 mL box)	115.0	106.5	−(7.4)	125.5	105.4	−(16.0)	104.2	96.7	−(7.2)	125.1	126.6	+(1.2)
Flavored milk (200 mL bottle)	85.3	77.1	−(9.6)	86.4	60.2	−(30.3)	83.4	89.4	+(7.2)	98.9	75.0	−(24.2)
Sour milk / yogurt (180 mL box)	81.3	76.4	−(6.0)	87.4	72.4	−(17.2)	77.6	79.4	+(2.3)	82.4	73.4	−(10.9)
Sour milk / yogurt (100 mL bottle)	51.6	54.0	+(4.6)	51.3	55.1	+(7.4)	51.5	52.2	+(1.4)	52.4	58.8	+(12.2)
Fresh-made iced tea / coffee (650 mL glass)	270.8	278.5	+(2.8)	230.8	249.6	+(8.2)	293.7	294.1	+(0.1)	218.9	253.0	+(15.6)
Fresh-made herbal drinks (500 mL glass)	179.4	185.9	+(3.6)	183.0	157.9	−(13.7)	178.0	185.3	+(4.1)	179.7	202.6	+(12.7)
Fresh-made smoothie juice (650 mL glass)	188.0	181.2	−(3.6)	188.3	167.5	−(11.0)	190.7	184.2	−(3.4)	173.9	197.9	+(13.8)
**Total**	**299.1**	**293.1**	**−(2.0)**	**337.1**	**332.6**	**−(1.3)**	**320.6**	**310.3**	**−(3.2)**	**210.4**	**221.2**	**+(5.1)**
**Total (including taxed and non-taxed groups)**	**474.0**	**453.8**	**−(4.3)**	**675.2**	**642.3**	**−(4.9)**	**495.7**	**478.6**	**−(3.4)**	**263.3**	**262.0**	**−(0.5)**

**Table 4:** Significance of the difference in ratio of average taxed to non-taxed SSBs consumed between 2018 and 2019, by sociodemographic characteristics (*n* = 5594).

Variables	*n* (%)	2018				2019				*t*-Value	*p*-Value
		Average Taxed Group (mL)	Average Non-Taxed Group (mL)	Mean (Ratio: Taxed Group/Non-Taxed Group)	SD	Average Taxed Group (mL)	Average Non-Taxed Group (mL)	Mean (Ratio: Taxed Group/Non-Taxed Group)	SD		
Total		**283.6**	**299.1**	**1.144**	**3.990**	**275.8**	**293.1**	**0.950**	**2.782**	**3.248**	**0.001**
**Sex**											
Male	2042 (36.5)	335.6	292.5	1.624	4.425	311.8	278.2	1.320	3.248	2.815	0.005
Female	3552 (63.5)	259.8	302.6	0.865	3.686	248.1	301.2	0.735	2.446	1.845	0.065
**Age (Years)**											
6–14	945 (16.9)	381.5	337.1	2.358	4.316	361.4	332.6	2.023	4.211	1.739	0.082
15–59	3071 (54.9)	282.2	320.6	1.191	4.691	280.8	310.3	0.973	2.864	1.767	0.077
60 or over	1578 (28.2)	160.3	210.4	0.444	1.550	148.8	221.2	0.267	0.890	4.286	<0.001
**Marital Status**											
Single	1604 (28.7)	379.4	356.4	2.062	5.065	368.5	338.7	1.698	3.753	2.488	0.013
Married	3250 (58.1)	232.4	272.5	0.816	2.280	221.3	276.2	0.706	2.164	2.258	0.024
Widowed/divorced/separated	740 (13.2)	185.1	257.0	0.684	6.145	195.1	248.0	0.480	2.490	0.878	0.380
**Place of Residence**											
Rural	2968 (53.1)	291.1	319.5	1.057	4.421	288.1	310.6	0.859	2.647	2.204	0.028
Urban	2626 (46.9)	275.4	275.3	1.242	3.436	263.0	273.9	1.052	2.924	2.471	0.014
**Region**											
Bangkok	854 (15.3)	326.8	337.4	1.161	2.561	321.6	304.0	0.829	2.421	2.867	0.004
Central	929 (16.6)	346.5	265.0	1.762	5.637	321.1	267.6	1.433	3.800	1.681	0.093
North	986 (17.6)	240.3	284.7	0.774	2.321	228.3	253.8	0.737	2.248	0.392	0.695
Northeast	1440 (25.7)	275.2	243.0	1.484	5.502	279.5	250.7	1.257	3.399	1.393	0.164
South	1385 (24.8)	241.5	359.3	0.628	1.713	237.3	372.9	0.532	1.378	1.842	0.066
**Educational Attainment**											
No formal education	764 (13.7)	285.1	308.8	1.286	3.034	277.9	277.8	1.181	3.522	0.705	0.481
Primary school	2839 (50.8)	257.6	261.5	0.982	2.674	259.2	268.1	0.819	2.625	2.539	0.011
Secondary school	1595 (28.5)	326.4	341.7	1.466	6.052	303.8	329.9	1.151	2.886	2.040	0.042
Bachelor’s or higher degree	396 (7.1)	268.2	354.4	0.669	1.684	241.9	321.6	0.591	1.410	0.786	0.432
**Income (Baht per Month)**											
No income	1883 (33.7)	347.9	322.1	1.866	5.633	344.0	327.5	1.547	3.747	1.908	0.057
Lower 10,000	2264 (40.5)	216.6	251.3	0.770	2.463	219.8	262.1	0.637	1.822	2.570	0.010
10,000–19,999	1214 (21.7)	275.4	342.6	1.109	4.292	281.6	309.0	0.936	3.100	1.264	0.206
20,000 and above	233 (4.2)	283.9	281.3	0.883	2.573	211.3	289.4	0.694	1.864	1.121	0.263
**Occupation**											
Unemployed	2649 (47.4)	314.7	300.6	1.289	4.413	312.7	301.1	1.073	3.065	2.172	0.030
Government	154 (2.8)	216.9	330.3	0.736	1.616	200.6	288.6	0.507	1.597	1.459	0.147
Company-hire	272 (4.9)	346.9	410.6	1.531	4.808	268.0	295.2	0.998	1.983	1.876	0.062
Own business	923 (16.5)	274.3	325.4	0.804	2.239	253.7	319.1	0.741	2.748	0.611	0.541
Wage laborer	600 (10.7)	293.3	282.3	1.543	5.925	309.7	305.4	1.290	3.403	0.974	0.331
Farmer	996 (17.8)	184.9	236.8	0.835	2.541	192.2	241.1	0.706	1.847	1.576	0.115
